# A Comprehensive Analysis into the Therapeutic Application of Natural Products as SIRT6 Modulators in Alzheimer’s Disease, Aging, Cancer, Inflammation, and Diabetes

**DOI:** 10.3390/ijms22084180

**Published:** 2021-04-17

**Authors:** Raushanara Akter, Afrina Afrose, Md. Rashidur Rahman, Rakhi Chowdhury, Saif Shahriar Rahman Nirzhor, Rubayat Islam Khan, Md. Tanvir Kabir

**Affiliations:** 1 Department of Pharmacy, Brac University, Dhaka 1212, Bangladesh; afrina.afrose@bracu.ac.bd (A.A.); chowdhuryrakhi888@gmail.com (R.C.); tanvir_kbr@yahoo.com (M.T.K.); 2 Department of Pharmacy, Faculty of Biological Science and Technology, Jashore University of Science and Technology, Jashore 7408, Bangladesh; mr.rahman@just.edu.bd; 3 Greehey Children’s Cancer Research Institute, The University of Texas Health Science Center at San Antonio, San Antonio, TX 78229, USA; nirzhor@livemail.uthscsa.edu; 4 Eppley Institute for Research in Cancer and Allied Diseases, University of Nebraska Medical Center, Omaha, NE 68198, USA; rubayat.khan@unmc.edu

**Keywords:** natural products, SIRT6, modulators, polyphenols and flavonoids, peptides, fatty acid

## Abstract

Natural products have long been used as drugs to treat a wide array of human diseases. The lead compounds discovered from natural sources are used as novel templates for developing more potent and safer drugs. Natural products produce biological activity by binding with biological macromolecules, since natural products complement the protein-binding sites and natural product–protein interactions are already optimized in nature. Sirtuin 6 (SIRT6) is an NAD+ dependent histone deacetylase enzyme and a unique Sirtuin family member. It plays a crucial role in different molecular pathways linked to DNA repair, tumorigenesis, glycolysis, gluconeogenesis, neurodegeneration, cardiac hypertrophic responses, etc. Thus, it has emerged as an exciting target of several diseases such as cancer, neurodegenerative diseases, aging, diabetes, metabolic disorder, and heart disease. Recent studies have shown that natural compounds can act as modulators of SIRT6. In the current review, a list of natural products, their sources, and their mechanisms of SIRT6 activity modulation has been compiled. The potential application of these naturally occurring SIRT6 modulators in the amelioration of major human diseases such as Alzheimer’s disease, aging, diabetes, inflammation, and cancer has also been delineated. Natural products such as isoquercetin, luteolin, and cyanidin act as SIRT6 activators, whereas vitexin, catechin, scutellarin, fucoidan, etc. work as SIRT6 inhibitors. It is noteworthy to mention that quercetin acts as both SIRT6 activator and inhibitor depending on its concentration used. Although none of them were found as highly selective and potent modulators of SIRT6, they could serve as the starting point for developing selective and highly potent scaffolds for SIRT6.

## 1. Introduction

Natural products are structurally distinct compounds obtained from diverse sources such as plants, microorganisms, animals, insects, minerals, and marine organisms [1,2]. Approximately 60% of all available drugs have been directly or indirectly derived from natural products such as aspirin, digoxin, morphine, artemisinin, camptothecin, lovastatin, maytansine, paclitaxel, penicillin, reserpine, and silibinin. Natural compounds have long been used for the amelioration of disease symptoms as well as prevention of diseases, or the complete recovery from various disorders [2,3]. Natural products with diverse pharmacological properties play a crucial role by providing a novel lead template for drug discovery and development. They form the basis of a wide array of modern medicines produced in the pharmaceutical and biotechnological industries [2]. Due to possessing chemical, functional, and structural diversity, natural small molecules endow them as potential targets of a more comprehensive number of biomolecules, especially proteins [4]. Biological effects of natural products can be explained by their binding with biological macromolecules since natural products complement the protein-binding sites and natural product–protein interactions are already optimized in nature [2,5,6]. Thus, natural product-derived scaffolds serve as superior template for the lead optimization [7]. Using combinatorial chemistry and human metabolites has revealed that the drugs are more similar to naturally occurring metabolites; thus, natural products are better than the screening molecules due to their inherent bioactivity [8]. Another study reported that as compared with synthetic compounds and combinatorial libraries, natural products might not have side effects; they have a wider distribution of molecular properties, such as structural diversity, low molecular mass, and partition coefficient; they have more interaction with proteins, enzymes, and other biomolecules; they contain fewer heavy metals, and possess higher molecular rigidity [2].

Several key cell signaling pathways are attributed to mitogenic, cytotoxic, and genotoxic aberrations causing disease pathologies. All of these signaling pathways are regulated by natural products with significant bioactivities [3]. It has been reported that compounds such as anagyrine, cytosine, sparteine, and lupanine produce their biological responses through mimicking endogenous molecules that are involved in intercellular or intracellular signal transduction. Conversely, different organisms utilize similar molecules for identical purposes, for example, brassinolide, which is structurally similar to human growth regulating steroids is used for cell division and development in plants [1].

## 2. Sirtuin 6 (SIRT6) and Its Association with Different Diseases

**Figure 1:**
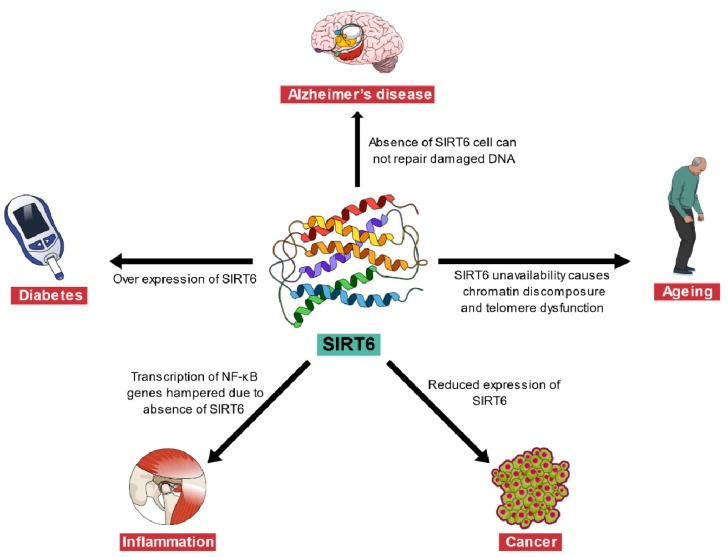
Sirtuin 6 (SIRT6) and its association with different diseases.

Sirtuin 6 (SIRT6) is a nicotinamide adenine dinucleotide+ (NAD+) dependent histone deacetylase enzyme and a unique member of the Sirtuin family. It displays histone 3 lysine 9 (H3K9), and histone 3 lysine 56 (H3K56) deacetylase, mono-ADP ribosyltransferase, and acylase activities, and these activities of SIRT6 are associated with the regulation of many genes [9,10]. SIRT6 also plays a crucial role in DNA damage signaling, DNA repairing, and is involved in metabolism. Researchers have discovered SIRT6 as a complex enzyme with several substrates and catalytic functions [11]. Furthermore, SIRT6 is also known to alter adult hippocampal neurogenesis through its influence on the number of glial and neuronal cells, thereby, offering a therapeutic arc in Alzheimer’s disease [12]. Thus, it has emerged as an exciting target of several diseases such as neurodegenerative diseases, aging, diabetes, cancer, metabolic disorder, and heart disease [13,14,15]. Table 1 and Figure 1 show the association of SIRT6 with different diseases such as Alzheimer’s disease, aging, cancer, inflammation, and diabetes.

### 2.1. Association of SIRT6 with Alzheimer’s Disease and Aging

Alzheimer’s disease (AD) is one of the most common and devastating neurodegenerative diseases. There are several proposed mechanisms implicated in its progression including, but not limited to, lysosomal storage, the effect of glial cells, and aggregation of tau proteins [16,17,18]. DNA damage accumulation has been found to be higher in brains affected by AD. SIRT6 has been reported to impact brain aging and neurodegenerative diseases such as AD [12,14,19]. Cells without SIRT6 cannot repair damaged DNA double-strand breaks and have insufficient base excision repairing capacity. SIRT6 repairs DNA damage recruiting chromatin remodeler SNF2H and deacylating H3K56 on those damaged sites. Studies have found that SIRT6 deficient mice showed more DNA damage; moreover, aged mice were characterized by reduced levels of SIRT6 [19].

Recent studies of genomes have found that chromatin discomposure and diminished repetitive DNA element silencing, such as silencing of retrotransposons, telomeres, and centromeres, are responsible for mammalian aging [11]. In fact, in aged tissues and cells, abnormal transcription and destabilization of these elements are observed [11,20]. SIRT6 has chromatin-regulatory activity at telomeres. In the Werner syndrome (premature aging disorder), deacetylation of SIRT6-dependent histone at telomeres produces a particular chromatin condition which is essential for DNA processing in the binding of ATP-dependent helicase of Werner syndrome (WRN) [11]. In the absence of SIRT6, telomere dysfunction results in genomic destabilization of primary human fibroblast and premature cell senescence [9]. Destabilization of repressive heterochromatin allows transcription of telomere-proximal genes, and deregulation of these genes causes cellular changes in aging and SIRT6 does reverse this phenomenon by stabilizing the repressive heterochromatin at the sub-telomeric region through silencing transcription of such genes [11,21]. Studies have revealed that reduced SIRT6 activity is linked to impaired heterochromatin sustentation, which leads to a premature aging disorder called Hutchinson-Gilford progeria syndrome (HGPS) in humans [22]. Poly (ADP-ribose) polymerase 1 (PARP1) contributes to recognize DNA damage and facilitates the choice of repair pathways, and SIRT6 is found to activate PARP1 when DNA damaged is occurred by oxidative stress [23].

SIRT6 provides more demyristoylation activity on peptide substrates as compared with deacetylation activity, but it catalyzes heavy histone deacetylation on whole chromatin and nucleosomes [24,25]. SIRT6 is effective in metabolism and DNA homeostasis; this has been demonstrated by studies that have shown extended lifespan of male mice through attenuated aging [26]. Therefore, activation of pharmacologic SIRT6 is beneficial for aging disorders. Apart from the deacetylation activity, SIRT6 also functions as a mono-ADP-ribosyltransferase. SIRT6 deacetylates lysine, which then gets coupled with NAD+ hydrolysis that produces nicotinamide and O-acetyl-ADP ribose [27,28]. This reaction is meant to allosterically inhibit the function of SIRT6 due to the production of nicotinamide. SIRT6 works at the molecular level to control the apoptosis, metabolism, aging, development, stress tolerance, and inflammation as a mono-ADP-ribosyl transferase-mediated by the SIRT6 gene in mammals [29]. SIRT6 is released from the nucleoli and is usually linked to the heterochromatic cell areas [30]. The highest levels of SIRT6 have been found in the liver, brain, thymus, muscles, and heart, where SIRT6 influences tissue-specific transcriptional regulation [31]. SIRT6 facilitates tolerance to DNA degradation and oxidative stress [29]. SIRT6 dysfunction or SIRT6 inactivation contributes to shortening lives and degenerative phenotypes in mice [32]. Deficiency of SIRT6 in mice showed severe age-related abnormalities such as loss of subcutaneous fat and extreme lymphopenia [33,34]. Compared to wild-type SIRT6 (WT), SIRT6-knockout (KO) mice appear normal in body weight at birth. However, within two weeks, metabolic/degenerative symptoms, including severe hypoglycemia, lymphocytic apoptosis, and wastage, are identified. Although the lymphocyte count is regular at conception, lymphocytes are destroyed in a systemic reaction in the process of apoptosis at about three weeks of age. Finally, the mice with SIRT6KO die at about four weeks. Cells of SIRT6KO have genetic instability, hypersensitive disruption to DNA, and other anomalies in their physiology [29,35]. SIRT6-specific knockdown cells (SIRT6 KD) are exposed to preterm cellular senescence and telomere disruption, as well as end-to-end chromosome fusion, where the degree of SIRT6 expression is decreased [36].

### 2.2. Association of SIRT6 with Cancer

SIRT6 can act as both a cancer promoter and a cancer suppressor. The reduction of SIRT6 expression is linked to the progression of different types of cancers such as colorectal, breast, ovarian, hepatocellular, and lung cancers. It has been exhibited that SIRT6 promotes tumor suppression inhibiting the hypoxia-inducible factor-1α (HIF-1α), which prevents the glycolytic metabolism of cancer cells [37,38]. Downregulation of SIRT6 expression has been detected in liver cancer, whereas SIRT6 is found to be overexpressed in chronic lymphocytic leukemia (CLL) in patients with liver cancer and CLL, respectively [38]. Besides, SIRT6 modulation has been suggested to play a pivotal role in the tumor microenvironment of various cancers [39,49]. SIRT6 overexpression has been found to be associated with prostate cancer and skin cancer [40].Downregulation of the lethal-7 (let-7) family of miRNAs suppresses myc target oncofetal proteins Lin28 and Lin28b, which cause SIRT6 knockdown and contribute to progression and metastasis of mouse and human pancreatic ductal adenocarcinoma (PDAC)[40]. SIRT6 suppression is regulated via the c-Fos pathway in hepatic cancer. Inhibition of histone H3K9 acetylation and nuclear factor kappa B (NF-κB) activation results in suppression of survivin, and thus c-Fos induces SIRT6. Upregulation of SIRT6 arrests cancer development through survivin’s anti-apoptotic activity [37]. Studies have also reported tumor-promoting activity of SIRT6. SIRT6 provides its oncogenic effect through chromatin remodeling. Deacetylation of H3K9 by SIRT6 inhibits B-cell lymphoma 2 (Bcl-2)-associated X protein (Bax) transcription; thus, it increases p53 gene and E2F-1 transcription factor chromatin accessibility, and eventually, apoptosis is inhibited [37]. Since SIRT6 plays a significant role in tumorigenesis, SIRT6 specific modulators with therapeutic and chemo-preventive potential would be of great interest in pharmacology [50].

### 2.3. Association of SIRT6 with Inflammation

SIRT6 also plays an important role in the modulation of immune reactions and inflammatory suppression throughout different parts of the human body. SIRT6 can regulate several NF-κB gene transcriptions both directly and indirectly [29]. SIRT6 deficiency enhances inflammatory response in adipose tissues, pancreatic β-cells, and endothelial cells of fat-specific Sirt6 knockout (FKO) sensitized mice [41]. Fibrosis and inflammation of liver have occurred in mouse immune cells [42]. NF-κB signal deactivation leads to a reduction in proinflammatory cytokines and anti-apoptotic gene generation [43]. SIRT6 directly influences a promoter of the NF-κB expression. SIRT6 alters tumor necrosis factor-α (TNFα) levels by monitoring post-translation rearrangement and involves the synthesis of interferon gamma (IFNγ). Such regulatory mechanisms provide an essential route to managing inflammatory cytokines associated with cell-mediated immunity and inflammation, making the nicotinamide phosphoryl transferase (Nampt)-SIRT6 axis. As a result, SIRT6 inhibitors may affect this regulatory pathway [29,43]. Another study showed that SIRT6 has a role in promoting the expression of proinflammatory cyto-/chemokines such as interleukin-8 (IL8) and tumor necrosis factor (TNF); these results confirmed the role of it in the expression of proinflammatory cytokines [14].

### 2.4. Association of SIRT6 with Diabetes

SIRT6 has recently received considerable attention since several studies have proven its critical roles in metabolism. SIRT6 controls regional and systemic energy metabolism and insulin resistance in major insulin-producing and insulin-target tissues such as adipose tissue, pancreatic β cells, skeletal muscle, and kidney [51]. SIRT6 can be regarded as an attractive target in the metabolic activity of glucose in light of animal studies conducted to develop new and efficient antidiabetics. In a previous study, it has been seen that mice with SIRT6 deficiency showed elevated uptake of glucose by tissue, high expression of glucose transporter glucose transporter 1 (GLUT1), and thus produced hypoglycemia [44,46]. Increased glycolysis and mitochondrial respiration suppression lead to high glucose consumption without SIRT6 [23]. An important glycolytic regulator, HIF1-α, modulates many genes such as lactic acid dehydrogenase (LDH), triose-phosphate isomerase (TPI), GLUT1, pyruvate dehydrogenase kinase 1 (PDK1), pyruvate dehydrogenase kinase 4 (PDK4), phosphofructokinase 1 (PFK1) either to increase glycolytic flux or to prevent mitochondrial respiration. It is noteworthy to mention that SIRT6 has been found to interact with this key glycolytic regulator HIF1-α and corepress it [45]. Therefore, the removal of SIRT6 enhances the expression of glycolytic enzymes and glucose transporters with HIF1-α mediated transcription [44]. Thus, antidiabetic agents may target SIRT6 inhibition that, in return, may cause glycolysis and reuptake of glucose. SIRT6 has also been observed to regulate gluconeogenesis; reduced glucose production is linked to reduced SIRT6 levels. Decreasing levels of SIRT6 deactivate general control non-repressed protein 5, which is responsible for increased acetylation levels of peroxisome proliferator-activated receptor-gamma coactivator 1 alpha (PGC-1α); thus, PGC-1α decreases the expression of gluconeogenic genes [44,47]. SIRT6 plays a potential role in regulating glucose since its deficiency leads to hypoglycemia by increasing insulin signaling and activating protein kinase, protein kinase B (Akt) [43]. SIRT6 in pancreatic β-cells deacetylates the forkhead box protein O1 (FoxO1) protein which in turns enhances the expression of two genes, i.e., pancreatic and duodenal homeobox 1 (Pdx1) and glucose transporter 2 (Glut2) to regulate the glucose sensing ability of pancreatic β-cells and systemic glucose tolerance [48].

## 3. Natural Products as Modulators of SIRT6: Promising Therapeutic Targets

Due to the significant implications of SIRT6 deacetylation, discovering activators and inhibitors of this enzyme has become a priority. SIRT6 inhibitors are found to inhibit cancer cell growth and promote apoptosis [14,52]. SIRT6 activators effectively treat diabetes and promote longevity [41]. SIRT6 action regulation compounds are thought to be potential drugs for age-related diseases, including obesity, diabetes, metabolic disorders, and neurological diseases [52]. Natural compounds with significant potency, isoform selectivity, and drug-like properties are needed to be identified to find SIRT6 as a validated target, and recently several natural compounds have been reported to modulate SIRT6 activity effectively [52,53]. In this current review, a number of natural activators and inhibitors, which belong to different phytochemical classes (Table 2 and Table 3), that can modulate SIRT6 are discussed with a focus on sources, mechanism of action, and role in SIRT6 modulation.

### 3.1. Natural Products Acting as SIRT6 Activators

#### 3.1.1. Polyphenols and Flavonoids

Natural polyphenols are plant-derived secondary metabolites with diverse pharmacological potentials [52]. Flavonoids belong to the polyphenol group act via different pathways and mechanisms involved in cancer, neurodegenerative diseases, diabetes, aging, metabolic syndrome, and inflammation, thus helping cure them. They are found to exert their beneficial effects in disease prevention [54]. Oxidative stress generates reactive oxygen species (ROS), and ROS are responsible for neurodegenerative diseases such as AD and aging [55]. Flavonoids exert their antioxidant effect by inhibiting enzymes involved in the free radical formation and subsequently suppress the generation of ROS [52]. SIRT6 regulates many stress responding genes by deacetylating histone 3 lysine 9 (H3K9) and histone 3 lysine 56 (H3K56) and through its mono-ADP ribosyltransferase and deacylase activities. A deficiency of SIRT6 in cells causes sensitivity to oxidative stress, and thus the DNA repair ability of the cells is reduced. Moreover, underexpression of SIRT6 in knockout mice has shown many early hallmarks of aging, whereas overexpression of SIRT6 extended the life span of the knock-out mice as compared with their counterpart [52]. Several polyphenols and flavonoids that act as SIRT6 activators are listed in Table 2 and are discussed in the following subsections.

##### Quercetin

Quercetin is a naturally occurring flavonoid found in onions, shallots, broccoli, peppers, caper fruits, apples, berries, grapes, herbs, tea, and wine (Table 2) [56]. Quercetin has been shown to have anti-inflammatory and antidiabetic activity. Quercetin has been reported to generate anticancer effect by inhibiting tyrosine kinase enzyme in vivo. It produces antidiabetic effect in patients with type 2 diabetes by ROS scavenging, thus, improving the antioxidant status in type 2 diabetic patients. Quercetin is used to treat chronic oral inflammation (oral lichen planus because it can restrict cytokines such as IL12, IL8, INFγ, INFα, cyclooxygenase 2 (COX-2), and prostaglandin E (PGE) [53]. A recent mass spectrometry (MS) study has identified the activating effect of SIRT6 at a high concentration of quercetin [57]. Quercetin has also been shown to increase the activity of SIRT6 at high concentrations in another study conducted by high performance liquid chromatography (HPLC)-employed SIRT6 assay [58]. Quercetin was reported to activate SIRT6 in the deacetylating genes H3K18ac and H3K9ac in nucleosome and on free full-length histone, respectively [57]. The H3K18ac gene is a potential target for anticancer therapy since this gene is a marker of cancer progression [59]. Recently, to discover the mechanism of SIRT6 activity modulation, analysis of binding and activity efforts of quercetin-based compounds on SIRT6 and other sirtuin isoforms were studied by You et al. They found that quercetin activates SIRT6 induced deacetylation by binding to the SIRT6-selective acyl binding channel [57]. Furthermore, the mechanism of quercetin-induced modulation of SIRT6 can be explained by its binding with of a large Rossmann fold domain and a small zinc-binding domain [60]. It was also reported that the specific small Zn^2+^ binding domain and cofactor binding loop provide SIRT6′s substrate acyl binding channel [57]. Ravichandran et al. studied the pharmacophore model of the binding site of SIRT6 protein for quercetin and their study generated a preliminary pharmacophore of the quercetin binding site on the SIRT6 protein that contained three hydrogen bond donors and one hydrogen bond acceptor [61].

##### Isoquercetin

Isoquercetin is also a naturally occurring flavonoid available in a vast number of natural sources such as medicinal herbs, fruits, beverages, vegetables, onions, mangoes, Tartary buckwheat bran, and Chinese hawthorn fruits (Table 2) [62,63,64,65]. It acts as a selective SIRT6 activator since it only binds to the SIRT6-selective acyl binding channel through its ability to distinguish between the SIRT6-selective acyl binding channel and an alternative binding site at the entrance of the active binding site of SIRT6. Furthermore, an isoform selectivity study of isoquercetin revealed that the bulky sugar moiety accommodates SIRT6’s acyl channel [57]. Isoquercetin has been reported to exert its cytotoxic/anticancer activity by inhibiting protein kinase B phopsphorylation, and thus surviving protein activates caspases and reduces anti-apoptotic proteins, i.e., Bcl-2 and Mcl-1 [63,66]. Another study has reported isoquerctein’s potential to produce antioxidant activity through scavenging of ROS [66]. The anti-inflammatory effect of isoquercetin is attributed to its regulation of Nrf2 pathway-associated protein and gene [67]. Isoquercetin also exerts antidiabetic activity and the mechanism involves reduction of oxidative stress, and also regulation of proteins and genes that are associated with nuclear factor erythroid 2-related factor 2 (Nrf2) [65]. All these bioactivities of isoquercetin may be linked to activation of SIRT6-induced deacetylation.

##### Kaempferol

Kaempferol is a flavonoid naturally occurring in green leafy vegetables such as spinach, kale, and herbs, dills, chives, tarragon, wild leeks, and ramps (Table 2) [68]. It has been found to be a weak SIRT6 activator in immunoblotting assay, whereas it was identified as a potent activator in the fluorogenic-based assay. A pharmacophore modeling revealed that kaempferol activates SIRT6 by binding to the SIRT6-specific acyl binding channel [61]. To determine the influence of kaempferol on the deacetylation activity of SIRT6, H3 peptide sequence was used as a substrate that simulated the biological deacetylation site of H3K56 in a fluorogenic assay. In contrast, core histone with full-length H3 peptide has been employed as a substrate in an immunoblotting assay. Therefore, the deacetylation activity of SIRT6 is substrate specific [13]. Several studies have reported on the different biological activities of kaempferol such as neuroprotective, antioxidant, anticancer, anti-inflammatory, and antidiabetic activity. Antioxidant capacity of kaempferol attributes to its ROS scavenging potential [69]. Neuroprotective effect in AD involves induction of anti-apoptotic activity in Aβ-induced SH-SY5Y neuronal cells [70]. Scavenging superoxide anions and hydroxyl radicals decreased peroxynitrile levels; inhibition of xanthine oxidase are responsible for its antioxidant effect [69]. Kaempferol exerts its anticancer effect through apoptosis, cell cycle arrest at the G2/M phase, downregulation of epithelial-mesenchymal transition (EMT)-related markers, and phosphoinositide 3-kinase/protein kinase B signaling pathways [69]. Kaempferol has also been reported to produce anti-inflammatory action by inhibiting the activity of of NF-κB and activator protein-1 (AP-1), and reducing gene expression of TNF-α, IL-1, and IL-8inhibiting activity as well [68,69].

##### Luteolin

Luteolin, a quercetin derivative that is found in carrots, peppers, celery, olive oil, peppermint, thyme, rosemary, lettuce, pomegranate, turnip, capers, cucumber, lemon, beets, brussels sprouts, cabbage, cauliflower, chives, fennel, hornwort, horseradish, kohlrabi, parsley, spinach, and green tea (Table 2) [57,71,72]. Rahnasto-Rilla et al. studied several flavonoids, including luteolin, by docking to find potent SIRT6 modulators, and they discovered that luteolin increased the deacetylation of SIRT6 at a high concentration [52]. In another study, luteolin produced increasing SIRT6 peptide deacetylation in a dose-dependent manner with a maximum two-fold stimulation [58]. Luteolin activates SIRT6 by binding with SIRT6-specific acyl binding channel [61]. Luteolin has anticancer, antioxidant, neuroprotective, anti-inflammatory, and other beneficial health effects. The neuroprotective effect of luteolin is due to scavenging of ROS [73]. This natural compound was also studied to reveal its impact on oxidative stress. The findings showed that it inhibited ROS production through the elevation of SIRT6 and other SIRTs FOXO3a expression [73]. Furthermore, the anti-inflammatory effect of luteolin is linked to its selective inhibition of COX-2, reduction of NFkappaB, and AP-1 activity [71,73]. Anticancer potential of any natural compound is of great importance and luteolin has this potential due to its inhibitory effect on topoisomerase I and II enzymes [71].

##### Cyanidin

Cyanidin is an anthocyanidin which can be found naturally in berries (bilberry, raspberry, and cranberry), black currant, and grapes [52,77]. Cyanidin plays a protective role in age-related diseases through the inhibition of ROS and nitrogen species production, thereby abrogating aging-related disorders [76,77]. Furthermore, ROS scavenging potential of cyanidin imparts its antioxidant effect [76]. Cyanidin has slightly increased SIRT6 activation potency and efficacy. The higher potency of cyanidin is due to an absence of the keto group at position 4 of the C-ring which eliminates unfavorable interaction with Met157 of SIRT6 [26]. In vitro study of cyanidin for its SIRT6 deacetylation activity has been conducted, and it has been found to be a potent SIRT6 stimulator with 55-fold maximum activity with an EC_50_ value of 460 ± 20 µM. Using Western blots, the SIRT6 deacetylation activity of cyanidin was estimated using core histone and the residual levels of histone H3 acetylation on lysine 9. Furthermore, cyanidin was found to upregulate SIRT6 associated gene, FOXO3α significantly, whereas it downregulated Twist1 and GLUT1 genes. The FOXO3α gene is involved in cell growth, proliferation, differentiation, and longevity, and more importantly, downregulation of the FOXO3α gene can result in a tumor. SIRT6 regulates FOXO3α by forming a complex with it, which as a result, upregulates the genes involved in antioxidation. Additionally, overexpression of the twist-related protein 1 (Twist1) and GLUT1 genes are associated with many tumors, and Twist1 is a crucial factor for tumor metastasis. SIRT6 suppresses cell proliferation through Twist1, and it also regulates metabolic cancer cell reprogramming through GLUT1 [52].

##### Fisetin

Fisetin is a bioactive flavonol which is abundantly found in fruits, vegetables, and medicinal herbs, including apples, grapes, persimmons, strawberries, cucumbers, and onions [79]. It has anticancer, antioxidant, antidiabetic, neuroprotective, anti-inflammatory, and other beneficial health effects [53,73]. Polyphenol fisetin is found to interfere with the molecular mechanisms involved in cell proliferation and cell death [38]. It has been reported to activate SIRT6. It has structural similarity with quercetin except for a hydroxyl group at the 5-C position [53]. Thus, fisetin may bind with SIRT6-specific acyl binding channel to activate SIRT6. This natural compound was studied to reveal its effect on oxidative stress. The result showed that it inhibited ROS production through the activation of SIRT6, other SIRTs, and reduction of FOXO3a gene expression [73]. Its anti-aging activity plays a crucial in the treatment AD patient and it exerts its anti-aging activity via reduction of Ckd5 activator p35 cleavage product, p25 in the brains of Alzheimer’s disease [53]. It also prevented the expression of cell adhesion molecules and suppressed NF-κB in vascular inflammatory responses [78]. Furthermore, fisetin has been reported to have anticancer activity against several cancer cells such as lung, colon, prostate, pancreas, and melanoma. The mechanism of fisetin’s anticancer action involves apoptosis, cell cycle arrest, suppression of Akt/mechanistic target of rapamycin (mTOR) signaling pathways, activation of caspase-7 and -9, inhibition of various key enzymes, and downregulation of several genes associated with cancers [53,79]. Another health benefit of fisetin is its antidiabetic effect and reduction of methylglyoxal dependent protein glycation has been evidenced as its mode of antidiabetic action [53].

##### Delphinidin and Its Derivative

Delphinidin is a member of anthocyanidins which is naturally found in flowers, fruits, vegetables, and grains [80,81,82,83,84]. SIRT6 modulation activity of delphinidin has been determined using core histones and estimating the remaining level of H3K9 by Western blot technique, and delphinidin was found to enhance SIRT6 deacetylation [52]. The structural basis of SIRT6 activation involves delphinidin’s binding to a binding site which is next to a loop near the acetylated peptide substrate binding site of SIRT6 and may cause conformational change of this loop [52]. Delphinidin-3-glycoside is present in the fruits of bog blueberry and has been reported to eliminate ROS, which resulted in the prevention of DNA damage responsible for p53 and Bad (Bcl-2 associated agonist of cell death) activation in UV-B-irradiated human dermal fibroblasts. Henceforth, it is capable of inhibiting skin pigmentation and photoaging [80]. Potent anti-aging action of delphinidin through scavenging of ROS and free radicals has also been reported [80,84]. Delphinidin and delphinidin-3- glycoside produced an anti-inflammatory effect through dose-dependent reduction of IL-1*β* and suppression of the NF-*κ* B pathway in vitro in a mechanistic study carried out by Murakami et al. [81]. Chen et al. reported that delphinidin ceased the proliferation of HER-2 positive breast cancer cells, MDA-MB-453 and BT474, and induced apoptosis and autophagy. In both breast cancer cells, delphinidin induced autophagy through mTOR signaling pathway suppression and AMP-activated protein kinase (AMPK) signaling pathway stimulation [83]. Significant free radical scavenging activity, anti-proliferative and apoptotic effects of delphinidin on MCF breast cancer cells have also been reported [83].

##### Icariin

Icariin is a prenyl flavonoid glycoside which is the primary active compound of *Herba epimedii.* It has been found to upregulate SIRT6 expression, decrease NF-κB protein expression, and inhibit anti-inflammatory response in mice [97]. It has been reported to exert anticancer, anti-inflammatory, antidepressant, and anti-aging effects [98,99]. Ovarian cancer cells (A2780) treated with different icariin concentrations inhibited cell proliferation, accelerated apoptosis, and caspase-3 activity by targeting phosphatase and tensin homolog (PTEN), reversion-inducing cysteine-rich protein with kazal motifs (RECK), and Bcl-2 protein expression in A2780 cells [99]. The anticancer effect of icariin was also studied in mouse Leydig tumors cells (MLTC-1). The findings revealed that icariin promoted cancer cell apoptosis by regulating the expression of Bcl-2/Bax and cytochrome c, activation of caspase-9, and -3 [100]. SIRT6, through its deacetylation activity, regulates Bax, which is the primary pathway involved in the apoptosis of cancer cells [101]. Furthermore, studies have also reported that anti-aging properties of icariin resulted in the inhibition of p53/p21 and NF-κB signaling pathways, and enhanced SIRT6 expression [85].

#### 3.1.2. Polysaccharides

##### Fucoidan

Fucoidan is a polysaccharide which is naturally found in seaweeds and brown algae [88]. It has been reported to possess antioxidant, anticancer, anti-inflammatory, antidiabetic, and immunomodulatory effects [88]. Fucoidans extracted from the *Sargassum filipendula* and *Laminaria japonica* has been shown to exhibit antioxidant activity greater than vitamin C [102,103,104]. A recent report mentioned that its antioxidant activity is attributed to higher scavenging of nitric oxide than the most widely used synthetic antioxidants, including butylated hydroxyanisole (BHA) and butylated hydroxytoluene (BHT) [88]. It was identified and isolated from *Fucus distichus* fungus and was investigated to determine its SIRT6 modulation activity and the results depicted a considerable increase in deacetylation activity of SIRT6 [58]. Researchers have reported that fucoidan showed SIRT6 specific action since it strongly enhanced SIRT6 deacetylation activity as compared with its SIRT1-SIRT3 isoforms deacetaylation activity. Furthermore, fucoidan-riched five microalgal extracts obtained from *Fucus distichu*s**, *Fucus vesiculous*, *Cytoseira tamariscofolia*, *Cytoseira nodacaulis*, and *Alaria esculenta* showed significant amplification of SIRT6 activity [58]. Most importantly, it exhibited dose-dependent SIRT6 stimulating activity [58]. Fucoidan induced apoptosis in HepG2 liver cancer cells through the upregulation of p53 and p14, as well as the stimulation of caspases activity. It is noteworthy to mention that overexpression of SIRT6 is associated with the induction of apoptosis in cancer cells. Furthermore, gluconeogenesis was inhibited by fucoidan through the stimulation of SIRT6 by p53, which in turn led to an enhanced level of FoxO1 [89]. It has also been reported that fucoidan provides antidiabetic action by inhibiting α-amylase and α-glucosidase. Moreover, the antidiabetic property of fucoidan is attributed to activate the PI3K/PKB pathway, which regulates insulin production and stimulates GLUT4 translocation [91]. Implications of fucoidan have also been identified in healing inflammation via inhibition of NO production, downregulation of iNOS, COX-2, IL-1 β, TNF-α, NF- κB expression, and regulation of ERK, JNK, MAPK, and Akt pathways [88,90].

##### Arabinoxylans

Arabinoxylans are present in cereal cell walls, wheat bran, rice bran, etc. [105]. A recent study reported that a polysaccharide, arabinoxylan-riched psyllium seed husk (PSH) upregulated SIRT6 expression in Sprague Dawley (SD) rats. Psyllium seed husk contains arabinoxylan 60 wt. % of PSH. The mechanism of SIRT6 stimulation by PSH might be the binding of pCREB to the binding site CRE on SIRT6 gene promoter ([106]. Arabinoxylan has been reported to produce immunomodulating and anticancer action in human immune cells (U937) and in gastric cancer cells ([105,107].

#### 3.1.3. Fatty Acids

The deacetylase function of SIRT6 has been endogenously activated by long-chain fatty acids [108]. The SIRT6 activator attachment on the distal portion of the fatty acyl substrate in the SIRT6 pocket has also been found with small molecules [26]. Deacetylation of SIRT6 has been induced with free long chains of fatty acids [95]. Studies have demonstrated that free fatty acid with divergent acyl chain length activates SIRT6 deacetylation by competitively inhibiting the peptide-conjugated myristoyl chain and occupying the hydrophobic pocket of SIRT6 [109]. Klein et al. investigated 64 fatty acids along with 190 bioactive lipids for initial targeted screening to identify selective SIRT6 activators, and they found that many of them stimulated SIRT6 with 5- to 12-fold activation [110]. They further identified a SIRT6 activator with more potency and selectivity through a SAR-guided chemical optimization study, where the activation of SIRT6 was mediated through the acceleration of catalytic step transpiring after substrate binding but before NAD+ cleavage [109]. Free fatty acids comprising 12 to 18 carbons were capable of enhancing SIRT6 deacetylation activity up to 35-fold [109]. One study found that, in SIRT6 knockout mice, intake of a high-fat diet could reverse metabolic disorders and premature aging, because fatty acids might be a better energy source to switch the physiological mechanisms [110]. SIRT6 can control circadian chromatin recruitment of SREBP-1, which regulates genes implicated in fatty acid metabolism. Hence, the SIRT6 knockout mice experienced disruption in fatty acid metabolism [111]. A novel SIRT6 activator, namely ginsenoside Rc, has been identified and ameliorated high fat diet-induced mitochondrial stress, oxidative stress, and inflammatory damage in mice [112].

##### Oleic Acid and Linoleic Acid

Oleic acid is found in plant-based oil (olive oil), nuts, vegetables [93], and linoleic acid is naturally available in plant-based oil, nut, meat, and animal products [96]. Both oleic acid and linoleic acid have been reported as potent activators of SIRT6. These long-chain fatty acids bind with a large hydrophobic pocket of SIRT6, and thus induce a conformational change that stimulates the deacetylation activity of SIRT6. Rahnasto-Rilla et al. studied a series of ethanolamides to identify whether they inhibit or activate SIRT6. They found that all these compounds exhibited a strong activating effect on SIRT6 at 100 µM concentrations with the fatty acids, thereby demonstrating a direct regulation relationship [60]. Nitro-oleic acid and nitro-conjugated linoleic acid bind to the hydrophobic slot of the SIRT6 active site causing a moderate activation of SIRT6 at 20 µM concentration [113]. Oleic acid and linoleic acid have been reported to produce free radical scavenging activity, thus, they might be effective in the treatment of oxidative stress-associated disorder [94].

##### Fatty Acid Derivatives (N-acylethanolamines)

It has been reported that myristoyl (MEA), oleoyl (OEA), and palmitoyl (PEA) ethanolamides resulted in the strongest SIRT6 activation, which was comparable to linoleic acid at the same concentration. However, at 10 µM concentration, linoleic acid was more effective as a SIRT6 activator than the ethanolamides of fatty acid [60]. The discovery of small molecules as SIRT6 activators demonstrates the potential of compounds that stimulate SIRT6 activity as anti-inflammatory and anti-tumorigenesis deacetylase. Feldman et al. studied the deacylase activity of SIRT1 to SIRT7, and they found that SIRT6 was very particular to deacylase the long-chain fatty acids such as myristoyl and palmitoyl chains. These fatty acid derivatives are expected to upregulate the SIRT6 activity, and therefore can be used as a therapeutic target for inflammation and cancer [95]. Degn, M., et al. investigated the changes in brain level of N-acylethanolamines in focal cerebral ischemia in mice and they found neuroprotective potential of these compounds [92].

### 3.2. Natural Products Acting as SIRT6 Inhibitors

Several polyphenols and flavonoids, fatty acid, and their derivatives that act as SIRT6 inhibitors are listed in Table 3 and are discussed in the following subsections.

#### 3.2.1. Polyphenols and Flavonoids

##### Quercetin, Its Derivatives and Luteolin

Rahnasto-Rilla et al. showed the SIRT6 modulating activity of several natural compounds, where quercetin and luteolin at lower concentration inhibited SIRT6 deacetylation activity [52,60]. Quercetin and luteolin provide their SIRT6 inhibitory effect by binding with an alternative site found on the entrance of an active site of SIRT6 [26]. Singh et al. examined several flavonoids such as quercetin, luteolin, vitexin, kaempferol, apigenin, and naringenin to screen their SIRT6 modulating potential and they reported quercetin as a SIRT6 inhibitor [114]. Heger et al. investigated the SIRTs modulating the activity of a set of polyphenols by in vitro study and molecular docking where some of the polyphenolic compounds were isolated from the root bark of *Morus nigra.* Among them, two quercetin derivatives, diquercetin and 2-chloro-1,4-naphthoquinone quercetin, were reported as promising SIRT6 inhibitors with the IC_50_ values of 130 and 55 µM, respectively. An interaction study of these polyphenolics with SIRT6 identified that diquercetin binds with the binding site of nicotinamide moiety (NAM), whereas 2-chloro-1,4-naphthoquinone-quercetin binds to the substrate-binding site of SIRT6 [13]. Diquercetin has been reported to produce promising antidiabetic effect by aldose and α-glucosidase activity inhibition. Flavonoids induce apoptosis in cancer cells through the Ca^2+^ -dependent mitochondrial pathway as well as through the inhibition of sarco/endoplasmic reticulum Ca^2+^-ATPase (SERCA). It was also been reported that some quercetin derivatives stimulate SERCA activity; thus, modulation of SERCA activity can be an effective approach to treat cancer and diabetes as these diseases are caused by SERCA activity impairment and disruption of Ca^2+^ balance in the body. Most importantly, SIRT6 regulates Ca^2+^ homeostasis by regulating the production of Ca^2+^ mobilizing nucleotides [115].

##### Vitexin

Vitexin is an apigenin flavone glycoside naturally available in hawthorn berry, mung beans, bamboo, buckwheat, echinodorus, and passiflora [116]. It is a primary polyphenolic compound of mung beans [117]. Singh et al. studied vitexin along with other flavonoids to screen their SIRT6 modulating potential, and they reported vitexin as a SIRT6 inhibitor [114]. Inhibitors belong to flavonoid mainly bind with a site close to the binding site of nicotinamide (NAM) moiety of NAD+ [52]. In addition, pharmacophore mapping has revealed that vitexin’s bulky sugar moiety poses a strain in the mapping of hydroxyl bond acceptor and reduces interaction with hydroxyl bond donor in the receptor which changes binding mode of vitexin [61]. In another study, vitexin at 10, and 100 µM concentrations were found to decrease ROS level and increase glutathione (GSH) and superoxide dismutase (SOD) levels, thus producing antioxidant effects and it was also evidenced as effective in the treatment of neurological disorders. Vitexin’s neuroprotecitve effect is attributed to downregulation of HIF1-α and VEGF and maintenance of blood brain barrier (BBB) integrity. Vitexin provides neuroprotection in AD by attenuating brain edema [53]. It has also shown antioxidant and antiapoptotic activity on glutamate-induced neurotoxicity in neuro-2a cells [118]. SIRT6 regulates key oxidative stress genes and mechanisms; and the expression and functions of SIRT6 aid in ROS reduction, and thus help in the treatment of ROS-induced diseases [119]. Vitexin generated an anticancer effect against several cancer cells including T24 bladder cancer and cell lung cancer A549 cells through the induction of apoptosis and regulation of expression of apoptosis-linked p53 and Bcl-2 genes. [53,116,120]. Several studies have reported the anti-inflammatory effect of vitexin and this effect was produced by different mechanisms such as inhibition of proinflammatory cytokines, i.e., IL-1β, IL-6, IL-8, IL-17, and IL-33, TNF-α, and inhibition of iNOS enzyme activation [53].

##### Catechin and Its Derivatives

Catechin is a polyphenolic compound naturally found in various foods and herbs, including tea, apples, persimmons, cacaos, grapes, and berries [121]. It showed significant inhibition of SIRT6 activity when the rational structure-activity relationship(SAR) of several polyphenolic compounds were studied to determine their SIRT6 histone deacetylation activity modulation. Catechin, epicatechin (cis stereoisomer of catechin), and (-) gallocatechin were studied to find their inhibition or activation potency against SIRT6 and the study revealed that the catechin (trans isomeric form) was the most potent among them as SIRT6 inhibitor [52]. Catechins are primary components of green tea, and they are found to have a cytoprotective effect against oxidative stress and inhibit DNA damage [122]. Henceforth, natural polyphenols that can regulate the activity of SIRT6 can be promising therapeutics for treating neurodegenerative diseases such as AD, aging, cancer, diabetes [52]. The structural basis of SIRT6 inhibition by small molecules was studied by molecular dynamics (MD) simulation and found that hydrophobic and π-stacking interactions are crucial for inhibition of SIRT6. Configuration matching is also important for SIRT6 inhibition [123].

##### Scutellarin

Scutellarin is a natural flavone compound found in the Chinese herb Huang Qin. It was studied by Shuang Zhao et al. to find its interaction with SIRT6 and was found to inhibit SIRT6 activity through hydrophobic and, potentially, π-stacking interaction. The configuration of scutellarin matching with SIRT6 also played a vital role in SIRT6 inhibition. It is worth mentioning that the Chinese people use this herb to cure cancer. Thus, the SIRT6 inhibitory effect of scutellarin may contribute to its chemotherapeutic potential [123]. This compound was also reported to have antioxidative activity along with anti-inflammatory properties. Oxidative stress inhibition was assessed by measuring the level of ROS, malondialdehyde (MDA), SOD, and GSH activity where ROS and MDA levels were found to decrease and SOD and GSH levels were found to increase. In addition, it has been reported that scutellarin has a role in promoting the activation of the Janus kinase 2 (JAK2)/signal transducer and activator of transcription 3 (STAT3) signaling pathway [124]. As scutellarin has a role in inhibiting oxidative stress and promoting the activation of the JAK2/STAT3 signaling pathway, targeting this compound might help in glioma treatment [125]. It has been reported that cisplatin-induced release of proinflammatory cytokines, TNF-α and IL-6, were suppressed by pretreatment with scutellarin in cisplatin-treated mice, while the anti-inflammatory effect produced by scutellarin was comparable to the positive control drug dexamethasone. Consequently, scutellarin demonstrated effective anti-inflammatory activity which was significant for the deterrence of cisplatin-induced renal damage [126].

#### 3.2.2. Vitamins

##### Nicotinamide

It is an amide form of vitamin B3 and is naturally found in eggs, meat, fish, and mushrooms [127]. Nicotinamide is a non-competitive inhibitor of SIRT6 and it reforms substrates by binding to the enzyme and reacting with the substrate-ADP-ribose adduct [109]. Studies have shown that nicotinamide hindered SIRT6 with an IC_50_ value of 184 μM. It blocked multiplication and advanced apoptosis in leukemic cells just as repressing the development and feasibility of human prostate cancer cells. It inhibited the proliferation while promoting apoptosis, specifically in leukemic and oral squamous cell carcinoma (OSCC) cells. SIRT6 acts as a tumor suppressor by promoting DNA repair and genome stability, glucose homeostasis, etc. [131]. SIRT6 knockout mice showed abbreviated life expectancy just as untimely maturing phenotypes, and also evidenced declined serum glucose and insulin-like development factor (IGF-1) levels [119]. Nicotinamide exerts its antidiabetic action by protecting the damage of pancreatic β-cells and preventing apoptosis and NO generation [128]. Previous studies have shown that nicotinamide is an inhibitor of SIRT6; therefore, suppressing nicotinamide can lead to SIRT6 protective effect against premature aging, hindering cancer growth, reducing the expression of IL8 and TNF as well as the reduction of serum glucose level [14,119].

#### 3.2.3. Peptides

Kokkonen et al. [132] investigated several peptides and pseudo peptides against SIRT1-SIRT6 and found that compound 5 was the most potent SIRT6 inhibitor with a 54% percentage rate. The longer peptide with the correct set of side chains was found more effective to bind with SIRT6. Thiomyristoyl peptides were studied to detect whether they can modulate SIRT6 or not. The finding revealed that thiomyristoyl peptides, mainly the BHJH-TM3 peptide, showed potent SIRT6 inhibition activity since it increased the lysine fatty acylation level of TNF-α. A tetrapeptide, i.e., chlamydocin, which was extracted from the organism *Diheterospora chlamydosporia* can also inhibit SIRT6 [133].

#### 3.2.4. Fatty Acid

##### Myristic Acid

Myristic acid is a naturally occurring fatty acid and abundantly found in milk fat, nutmeg, palm kernel, and butter [129,130]. Myristic acid has been shown to be a competitive inhibitor of SIRT6 demyristoylation where the free fatty acid (FFA) and peptide-conjugated myristoyl chain compete for the same hydrophobic pocket of SIRT6 protein [109]. To test the theory that myristic acid ties in a similar site as the unsaturated fat chain of a myristoylated lysine peptide, SIRT6 demyristoylase movement was estimated within sight of expanding myristic acid at a fixed peptide substrate concentration. In addition, a detailed steady-state analysis was performed to prove further that myristic acid and the fatty acid chain of a myristoylated peptide share the same binding site on SIRT6. This study clearly showed their competitive inhibition of SIRT6, as H3K9Myr peptide concentration was varied at several fixed concentrations of myristic acid [95]. Myristic acid had restrained the demyristoylase action of SIRT6 with an IC_50_ of 190 ± 10 μM. Previously there was a perception that SIRT6 demyristoylation was entirely unrelated to FFA; however, it has been proven that expanded cell levels of explicit fatty acids (FAs) trigger a change from SIRT6-subordinate demyristoylation to actuated histone deacetylation. SIRT6 attenuates NF-κB signaling through H3K9Ac deacetylation and also improves a supportive proinflammatory reaction by advancing the emission of TNF-α through lysine demyristoylation. Therefore, myristic acid plays a critical role as an inhibitor of SIRT6 by inhibiting inflammation [95]. Myristic acid is a competitive inhibitor of SIRT6. Thus, it might have anticancer activity since SIRT6 has essential capacities in cell procedures, such as gene expression, genome stabilization, and DNA fix [119].

## 4. Conclusions

Natural compounds possess an inherent ability to bind with biomolecules and provide diverse bioactivity such as anticancer, anti-inflammatory, antidiabetic, anti-aging, and antioxidant activity. Many natural products and their derivatives belong to different phytochemical classes such as polyphenols and flavonoids, polysaccharides, peptides, fatty acids, and have been identified as modulators of SIRT6. Sources of these compounds include various vegetables, fruits, grains, marine weeds, herbs, and medicinal plants. SIRT6 is a stress response protein deacetylase, and upregulation and downregulation of this protein has been associated with multiple disease processes. Studies have shown that some of the identified natural compounds can increase the deacetylation activity of SIRT6, and some have reported to decrease the deacetylation activity of it. Regular intake of these pure compounds or foods enriched with these compounds might be beneficial in the treatment of Alzheimer’s disease, aging, cancer, inflammation, and diabetes. However, the absolute potential to activate or inhibit SIRT6 in order to impart a therapeutic effect remains to be eluded. Thus, future research should have a vast scope to further characterize natural compounds and their derivatives with isoform selectivity, more potency, and more drug-like properties for the treatment of variegated diseases.

## Figures and Tables

**Table 1:** Association of SIRT6 with Alzheimer’s disease, aging, cancer, inflammation, and diabetes.

Disease	Molecular Mechanism of Disease	In Vitro/In Vivo Studies	Expression of SIRT6 Level	Role of SIRT6	References
Alzheimer’s disease	DNA damage	Study was conducted on mice	Deficiency or reduced level of SIRT6	SIRT6 repairs DNA damage recruiting chromatin remodeler SNF2H and deacylating H3K56 on those damaged sites	[12,14,19]
Aging	DNA damage, abnormal transcription and destabilization of retrotransposon, telomeres, and centromeres, dysfunction of telomeres, impaired heterochromatin sustentation	Study was carried out on aged tissues and cells of SIRT6 KO mice, human fibroblasts, and premature cells; SIRT6 KD cells	Absence or deficiency or decreased level of SIRT6	SIRT6 stabilizes repressive heterochromatin at the sub-telomeric region through silencing transcription of telomere proximal genes/activates PARP1/provides chromatin-regulatory activity/deacylates whole chromatin and nucleosomes	[11,21,22,26,27,28,31,32,34,35,36]
Cancer	Activation of HIF-1α factor, suppression of Lin28 and Lin28b oncofetal proteins, suppression of survivin protein, activation of Bax protein transcription	Studies conducted in patients with liver and chronic lymphocytic leukemia (CLL); patients, human cancer tissues or cells are studied	Downregulation in colorectal, breast, ovarian, hepatocellular, and lung cancers and p-regulation of SIRT6 in prostate, skin, CLL, and pancreatic cancers	SIRT6 inhibits the HIF-1α factor; activates Lin28, Lin28b, and survivin proteins; suppresses Bax protein transcription	[37,38,39,40,41,42]
Inflammation	NF-κB signal deactivation leads to a reduction in proinflammatory cytokines and anti-apoptotic gene generation, reduced expression of IL8 and TNF	FKO mice, mouse immune cells	Absence of SIRT6/deficiency of SIRT6	SIRT6 can regulate several NF-κB gene transcriptions, SIRT6 has a role in promoting the expression of proinflammatory cyto-chemokines such as interleukin-8 (IL8) and TNF	[14,29,41,42,43]
Diabetes	Decreased uptake of glucose by tissue, low expression of GLUT1; increased glycolysis and mitochondrial respiration suppression; increased level of HIF1-α, decreased acetylation levels of PGC-1 α; inhibition of protein kinase, Akt, low expression of Pdx1 and Gult2	Studies are conducted in β-specific KO mice	Enhanced level of SIRT6	Reduced level of SIRT6 upregulates GLUT1, Pdx1, and glut2; downregulates the level of HIF1-α; increases acetylation of PGC-1 α; stimulates protein kinase Akt, thus regulates glucose homeostasis	[43,44,45,46,47,48]

**Table 2:** Natural products acting as SIRT6 activators.

Compound (Class)	Source	Mode of Action against the Selected Diseases	Role in SIRT6 Activation	References
Quercetin (flavonoid)	Onions, shallots, broccoli, peppers, caper fruits, apples, berries, grapes, herbs, tea, and wine	Anticancer activity: Inhibition of tyrosine kinase in vivo, inhibition of PI3K (phosphatidylinisitol-3-kinase)-Akt-PKB (protein kinase B) pathway Antidiabetic effect: Scavenging reactive oxygen species (ROS) such as peroxynitrile, hydroxyl radicals, and superoxide anions Anti-inflammatory action: Prevention of cytokines including IL12, IL8, INFγ, INFα, COX-2, PGE	To activate SIRT6 quercetin binds to SIRT6-selective acyl binding channel and activation of SIRT6 provides all these bioactivities though different mechanisms	[56,74,75]
Isoquercetin (flavonoid)	Medicinal herbs, fruits, beverages, vegetables, onions, mangoes, Tartary buckwheat bran, Chinese and hawthorn fruits	Cytotoxic/anticancer activity: Inhibition of protein kinase B phopsphorylation, and thus surviving protein activates caspases and reduces anti-apoptotic proteins, i.e., Bcl-2 and Mcl-1 Antioxidant effect: Scavenging ROS Anti-inflammatory action: Regulation of Nrf2 pathway-associated protein and gene expression Antidiabetic effect: Reduction of oxidative stress, and also regulation of proteins and genes that associated with Nrf2 pathway.	Isoquercetin binds with SIRT6-selective acyl binding channel through its bulky sugar moiety to activate SIRT6 and activation of SIRT6 exerts these bioactivities	[56,61,63,64,65,66,67,74]
Kaempferol (flavonoid)	Green leafy vegetables such as spinach, kale, herbs, dills, chives, tarragon, wild leeks, and ramps	Neuroprotective effect in AD: Induction of anti-apoptotic activity in Aβ-induced SH-SY5Y neuronal cells. Antioxidant activity: Scavenging superoxide anions, hydroxyl radicals, decrease peroxynitrile levels, inhibition of xanthine oxidase enzyme. Anticancer potential: Mechanism involves apoptosis, cell cycle arrest at the G2/M phase, downregulation of epithelial-mesenchymal transition (EMT)-related markers, and phosphoinositide 3-kinase/protein kinase B signaling pathways. Anti-inflammatory effect: Inhibition of NF-κB activity and TNF activity, reduction of expression of IL-1 and IL-8, inhibition of the activation of AP-1, COX-2 Antidiabetic activity: Reduction of ROS level	To activate SIRT6 kaempferol binds to the SIRT6-specific acyl bonding channel and activation of SIRT6 results in these biological actions	[68,69,70]
Luteolin (flavonoid)	Carrots, peppers, celery, olive oil, peppermint, thyme, rosemary, lettuce, pomegranate, turnip, capers, cucumber, lemon, beets, brussels sprouts, cabbage, cauliflower, chives, fennel, harwort, horseradish, kohlrabi, parsley, spinach, and green tea	Neuroprotective action: ROS scavenging Antioxidant activity: ROS scavenging through elevation of SIRT6 expression Anticancer potential: Inhibition of topoisomerase I and II Anti-inflammatory action: Selective inhibition of COX-2, reduction of NFkappaB and AP-1 activity	Luteolin activates SIRT6 by binding with SIRT6-specific acyl binding channel. Stimulation of SIRT6 deacetylation activity contributes to these biological effects	[71,72,73]
Cyanidin (anthocyanidin)	Berries, black currant, grapes	Anti-aging property: Inhibition of ROS and nitrogen species production Anticancer activity: Upregulation of SIRT6 associated gene, FOXO3α; downregulation of SIRT6 associated Twist1 and GLUT1 genes; inhibition of epidermal growth factor receptor Antioxidant activity: ROS scavenging	To activate SIRT6 cyanidin binds with SIRT6-selective acyl binding site and in the same mood as quercetin. Stimulation of SIRT6 contributes to anti-aging, antiancer, and antioxidant effects	[52,76,77]
Fisetin (flavonoid)	Apples, grapes, persimmons, strawberries, cucumbers, and onions	Anti-aging property: Reduction of Ckd5 activator p35 cleavage product, p25 in the brains of Alzheimer’s disease patient Anticancer effect: Inhibition of Akt/mTOR signaling pathways, activation of Caspase-7 and-9 Antidiabetic activity: Reduction of methylglyoxal dependent protein glycation Antioxidant potential: Inhibition of ROS production Anti-inflammatory action: Suppression of NF-κB in vascular inflammatory responses	Structural basis of SIRT6 activation by fisetin is not well known, however, activation of SIRT6 brings these biological responses	[53,78,79]
Delphinidin (anthocyanidin)	Flowers, fruits, vegetables, and grains	Anti-aging action: ROS and free radical scavenging Anti-inflammatory activity: Reduction of IL-1*β* and suppression of NF-*κ* B pathway in vitro Anticancer effect: Induction of apoptosis, autophagy thorough mTOR signaling pathway suppression, and AMPK pathway activation	Delphinidin activates SIRT6 by binding to a binding site which is next to a loop near the acetylated peptide substrate binding site of SIRT6 and activation of SIRT6 results in anti-aging, anti-inflammatory, and anticancer effects	[80,81,82,83,84]
Icariin (prenylated flavonoid glycoside)	*Herba epimedii*	Anticancer potential: Inhibition of cell proliferation, accelerated apoptosis, and caspase-3 activity by targeting PTEN, RECK, and Bcl-2 protein expression in ovarian cancer cells (A2780); regulation of the expression of Bcl-2/Bax and cytochrome c, activation of caspase-9, and -3 in MLTC-1 mouse tumor cells. Anti-inflammatory effect: Reduction of NF-κB protein expression Anti-aging effect: Inhibition of p53/p21 and NF-κB signaling pathways, overexpression of SIRT6	Structural basis of SIRT6 activation by icariin is not well known, however, activation of SIRT6 brings all these biological responses	[85,86]
Fucoidan (polysaccharide)	Seaweeds and brown algae	Antioxidant effect: NO scavenging Anticancer potential: Induction of apoptosis in HepG2 liver cancer cells by upregulation of p53 and p14 and stimulation of caspases activity Anti-inflammatory action: Inhibition of NO production; downregulation of iNOS, COX-2, IL-1 β, TNF-α, NF- κB expression, ERK, JNK, MAPK, and Akt pathways. Antidiabetic effect: Stimulation of SIRT6 by p53, and enhancement of FoxO1 level. Activation of PI3K/PKB pathway, which regulates insulin production and stimulate GLUT4 translocation	Structural basis of SIRT6 activation by fucoidan is not well known, however, activation of SIRT6 brings several biological responses	[87,88,89,90,91]
N-acylethanolamines (NAEs) (lipid)	Endogenous molecules	Neuroprotection: Regulation of neuroprotection in focal cerebral ischemia in mice Anticancer and anti-inflammatory action: Upregulation of SIRT6	These compounds bind with a large hydrophobic pocket of SIRT6 and they stimulate SIRT6 to produce neuroprotection, anticancer, and anti-inflammatory effects	[60,92]
Oleic acid (fatty acid)	Plant-based oil (olive oil), nuts, and vegetable	Antioxidant activity: Free radical scavenging	Oleic acid stimulates deacetylation activity of SIRT6 by binding with a large hydrophobic pocket of SIRT6 and provides antioxidant activity	[60,92,93,94,95]
Linoleic acid (fatty acid)	Plant-based oil, nut, meat, and animal products	Antioxidant activity: Free radical scavenging	Same as oleic acid	[60,93,94,96]

**Table 3:** Natural products acting as SIRT6 Inhibitors.

Compound (Class)	Source	Mode of Action against the Selected Diseases	Role in SIRT6 Inhibition	References
Quercetin (flavonoid)	Onions, shallots, broccoli, peppers, caper fruits, apples, berries, grapes, herbs, tea, and wine	Anticancer and antidiabetic activity: Inhibition of SERCA (sarco/endoplasmic reticulum Ca^2+-^ATPase)	To inhibit SIRT6 quercetin binds with an alternative site on the entrance of active site of SIRT6 and inhibition of SIRT6 produces anticancer and antidiabetic effects	[56,115]
Vitexin (apigenin flavones glycoside)	Hawthorn berry, mung beans, bamboo, buckwheat, echinodorus, and passiflora	Antioxidant effect: Reduction of ROS level and enhancement of GSH and SOD level Neuroprotective effect in AD: Down egulation of HIF1-α and VEGF and maintenance of blood brain barrier (BBB) integrity, reduction of brain edema Anticancer activity: Apoptosis, regulation of apoptosis-related gene expression of p53 and bcl-2 Anti-inflammatory effect: Inhibition of IL-1β, IL-6, IL-8, IL-17, and IL-33, TNF-α, NFκ-B, and iNOS	Vitexin inhibits SIRT6 by binding to a site close to the NAM binding site of NAD+ and inhibition of SIRT6 contributes to therapeutic potential in different diseases	[52,53,61,116]
Catechin (polyphenol)	Variety of foods and herbs including tea, apples, persimmons, cacaos, grapes, and berries	Cytoprotective, anticancer, antidiabetic and neuroprotective effects: Reduction of oxidative stress and inhibition of DNA damage	Catechin inhibits SIRT6 by hydrophobic and π-stacking interactions and configuration matching which results in reduction of oxidative stress and DNA damage	[52,121,122,123]
Scutellarin (flavone)	*Scutellaria baicalensis*	Chemotherapeutic effect: Activation of JAK2/STAT3 signaling pathway Antioxidant activity: Reduction of ROS and MDA level, enhancement of SOD and GSH level Anti-inflammatory effect: Inhibition of proinflammatory cytokines, TNF-α, and IL-6 release	Scutellarin inhibits SIRT6 activity through hydrophobic and, potentially, π-stacking interaction, and thus provides chemotherapeutic, antioxidant and anti-inflammatory effects	[124,125,126]
Nicotinamide (amide form of vitamin B3-Niacin)	Eggs, meat, fish, and mushrooms	Anticancer activity: Apoptosis, DNA repairing, and genome stability Antidiabetic action: Induction of antidiabetic action by protecting pancreatic β-cells through prevention of apoptosis and NO generation Anti-aging: Reduction of ROS level	As a non-competitive inhibitor of SIRT6 nicotinamide reforms substrates by binding to the enzyme and reacting with the substrate-ADP-ribose adduct. Inhibition of SIRT6 is associated with various health benefits	[109,127,128]
Myristic acid (fatty acid)	Milk fat, nutmeg, palm kernel, and butter	Anti-inflammatory effect: Suppression of NF-κB signaling pathway and emission of TNF-α Anticancer potential: Genome stabilization, DNA repairing	Myristic acid inhibits SIRT6 competing with peptide-conjugated myristoyl chain for the same hydrophobic pocket of SIRT6 protein, and thus it plays a role in inhibiting inflammation and cancer	[109,129,130]

## Data Availability

Not applicable.
